# Comparison of linkage disequilibrium levels in Iranian indigenous cattle using whole genome SNPs data

**DOI:** 10.1186/s40781-015-0080-2

**Published:** 2015-12-24

**Authors:** Karim Karimi, Ali Esmailizadeh Koshkoiyeh, Cedric Gondro

**Affiliations:** Department of Animal Science, Faculty of Agriculture, Shahid Bahonar University of Kerman, Kerman, PB 76169-133 Iran; Young Researchers Society, Shahid Bahonar University of Kerman, Kerman, PB 76169-133 Iran; School of Environmental and Rural Science, University of New England, Armidale, NSW Australia

**Keywords:** Linkage disequilibrium, Single nucleotide polymorphism, Iranian indigenous cattle, Bovine genome

## Abstract

**Background:**

Knowledge of linkage disequilibrium (LD) levels among different populations can be used to detect genetic diversity and to investigate the historical changes in population sizes. Availability of large numbers of SNP through new sequencing technologies has provided opportunities for extensive researches in quantifying LD patterns in cattle breeds. The aim of this study was to compare the extent of linkage disequilibrium among Iranian cattle breeds using high density SNP genotyping data.

**Results:**

A total of 70 samples, representing seven Iranian indigenous cattle breeds, were genotyped for 777962 SNPs. The average values of LD based on the r^2^ criterion were computed by grouping all syntenic SNP pairwises for inter-marker distances from 0 Kb up to 1 Mb using three distance sets. Average r^2^ above 0.3 was observed at distances less than 30 Kb for Sistani and Kermani, 20 Kb for Najdi, Taleshi, Kurdi and Sarabi, and 10 Kb for Mazandarani. The LD levels were considerably different among the Iranian cattle breeds and the difference in LD extent was more detectable between the studied breeds at longer distances. Lower level of LD was observed for Mazandarani breed as compared to other breeds indicating larger ancestral population size in this breed. Kermani breed continued to have more slowly LD decay than all of the other breeds after 3 Kb distances. More slowly LD decay was observed in Kurdi and Sarabi breeds at larger distances (>100 Kb) showing that population decline has been more intense in more recent generations for these populations.

**Conclusions:**

A wide genetic diversity and different historical background were well reflected in the LD levels among Iranian cattle breeds. More LD fluctuation was observed in the shorter distances (less than 10 Kb) in different cattle populations. Despite of the sample size effects, High LD levels found in this study were in accordance with the presence of inbreeding and population decline in Iranian cattle breeds.

## Background

Linkage disequilibrium (LD) is defined as the non-random association of alleles at different loci within a population. Pattern of LD within a population can be affected by several factors including selection, mutation rate, migration, genetic drift, population structure and recombination rates [1]. Detection of genomic regions under selection pressures [2], exploring the genetic basis of economically important traits [3] and diversity between cattle breeds [4] can be investigated using comparison of LD maps. In recent years, genomic selection was successfully implemented in dairy cattle and is being developed to other livestock species. The basis of the genomic selection is the existence of LD between causative variants and genetic markers [5, 6]. Hence, efficient implementation of this method and delivery of accurate genomic predictions depend on the extent of linkage disequilibrium within a population [7]. Moreover, efficiency of some other routine studies applied to animal breeding such as genome-wide association studies (GWAS), genomic marker imputation, marker assisted selection (MAS), quantitative trait loci (QTL) mapping and parentage testing are impressed by levels of LD in studied populations [4, 8].

Depending on the population and the threshold used to measure LD, the average extent of LD is highly variable in different studies. Compared to human studies, likely due to smaller effective population size and intensive selection pressure, higher levels of LD have been found in livestock species [9–11]. Although, several studies have been conducted regarding linkage disequilibrium in cattle populations based on microsatellite markers [12, 13], availability of large numbers of SNP through new sequencing technologies has provided opportunities for extensive researches in quantifying LD patterns in cattle breeds [14–16]. Extensive LD along with different patterns on each chromosome observed in different cattle breeds, have confirmed that the LD maps can be used to characterize the cattle populations. Furthermore, since LD decays as a function of the number of generations, LD data have been frequently applied to estimate Ne at any particular time in the past in the cattle populations [17, 18]. Despite of particular LD characteristics of each population, results of these studies have been to some extent affected by some factors such as sample size, minor allele frequency (MAF) thresholds, density of SNP panels and distance between markers [19].

Cattle domestication and raising have an historical origin in Iran. Iranian indigenous cattle have been keeping in different geographical regions of the country and have been adapted with various environment conditions. Some important traits including resistance to local diseases and parasites (such as Theileriosis, Babesiosis and intestinal Nematodes), adaptation to low quality feed resources and heat tolerance were attributed to these breeds. In recent years, high dense SNP data has been widely applying as the standard tools in LD analysis of livestock populations. However, the level of LD has not been previously investigated in the Iranian indigenous cattle. Therefore, the objective of this study was to compare the LD levels among different Iranian cattle populations based on a high density SNP data set. Knowledge of difference in LD levels of these populations can help to detect diversity between cattle breeds and to investigate the historical changes in population sizes. Moreover, the applicability of the modern genomic technologies such as genomic selection and genome-wide association studies can be compared between different populations using the LD data.

## Methods

### Samples collection and genotyping

For this study, ten samples per each breed were collected from 70 individuals representing seven Iranian indigenous cattle breeds. Unrelated individuals were selected where possible based either on pedigree or farmers information. Animals used in this study were included the three main cattle types: Bos taurus breeds (Sarabi and Kurdi), Bos indicus (Sistani) and composite cattle (Taleshi, Mazandarani, Kermani and Najdi). Samples were genotyped using the BovineHD SNP chip (Illumina, Inc, San Diego, CA, USA) designed to genotype 777,962 SNPs.

### Quality control and minor allele frequency distribution

Quality control (QC) were performed using PLINK 1.07 software [20]. The SNPs located on X, Y and mitochondrial chromosomes (39367, 1224 and 343 SNPs, respectively) were excluded from the data set. The whole autosomal genome included 2612.82 Mb and the lengths of autosomal chromosomes ranged from 160.88 Mb (BTA1) to 43.08 Mb (BTA25). SNPs with MAF higher than 0.05 and with call rates of 90% or greater both by locus and by animal were selected. Also, SNPs deviating from Hardy-Weinberg equilibrium (HWE) at a *p*-value < 10^−7^ were removed from data set. MAF was calculated using PLINK for all autosomal SNPs and the distribution of the allelic frequencies was graphed as the proportion of the SNPs represented in 6 different categories of MAF: <0.05, ≥0.05 to <0.1, ≥0.1 to <0.2, ≥0.2 to <0.3, ≥0.3 to <0.4 and ≥0.4 to ≤0.5.

### Estimation of linkage disequilibrium

Among several proposed measures to estimate LD in a population, D′ [21] and r^2^ [22] are two statistic parameters widely used to measure the extent of LD. The r^2^ has been known as a more robust statistic due to less sensitivity to sample size and allele frequency differences [23]. The LD between two SNPs was evaluated using r^2^ defined as the correlation coefficient between SNP pairs, based on the following equation [24]:

1 $$ {r}^2 = \frac{{\left( freq AB* freq ab- freqAb* freq aB\right)}^2}{\left( freq\ A* freq\ a* freq\ B* freq\ b\right)} $$

In the above equation, freq A, freq a, freq B and freq b are the frequencies of alleles A, a, B and b, respectively, and freq AB,freq ab, freq aB and freq Ab are the frequencies of the haplotypes AB, ab, aB and Ab in the population, respectively. The measures of LD (r^2^) were calculated for all marker pairs of each chromosome (syntenic SNPs) using the SnppldHD software (Sargolzaei, M., University of Guelph, Canada). The r^2^ calculation was limited to the SNPs within the maximum distances of 15 Mb from each other. A sample size correction was performed on all of the computed r^2^ values using the below equation [25]:

2 $$ {r}^2\  corrected = \frac{r^{2\ } computed - \frac{1}{n}}{1 - \frac{1}{n}} $$

where, *n* is the number of haplotypes in the sample.

Average r^2^ between all adjacent SNPs was calculated for each breed. Maximum distances between syntenic SNP pairs was categorized as ≤10Kb, ≤100Kb, and ≤1Mb distances and for each distance category, SNP comparisons were binned using bin sizes of 1 Kb, 10 Kb, and 100 Kb, respectively. The mean r^2^ was computed for each bin in whole autosomal chromosomes. The mean r^2^ in each distance bin was plotted against the median of the distance bin range (Kb).

## Results

**Fig. 1:**
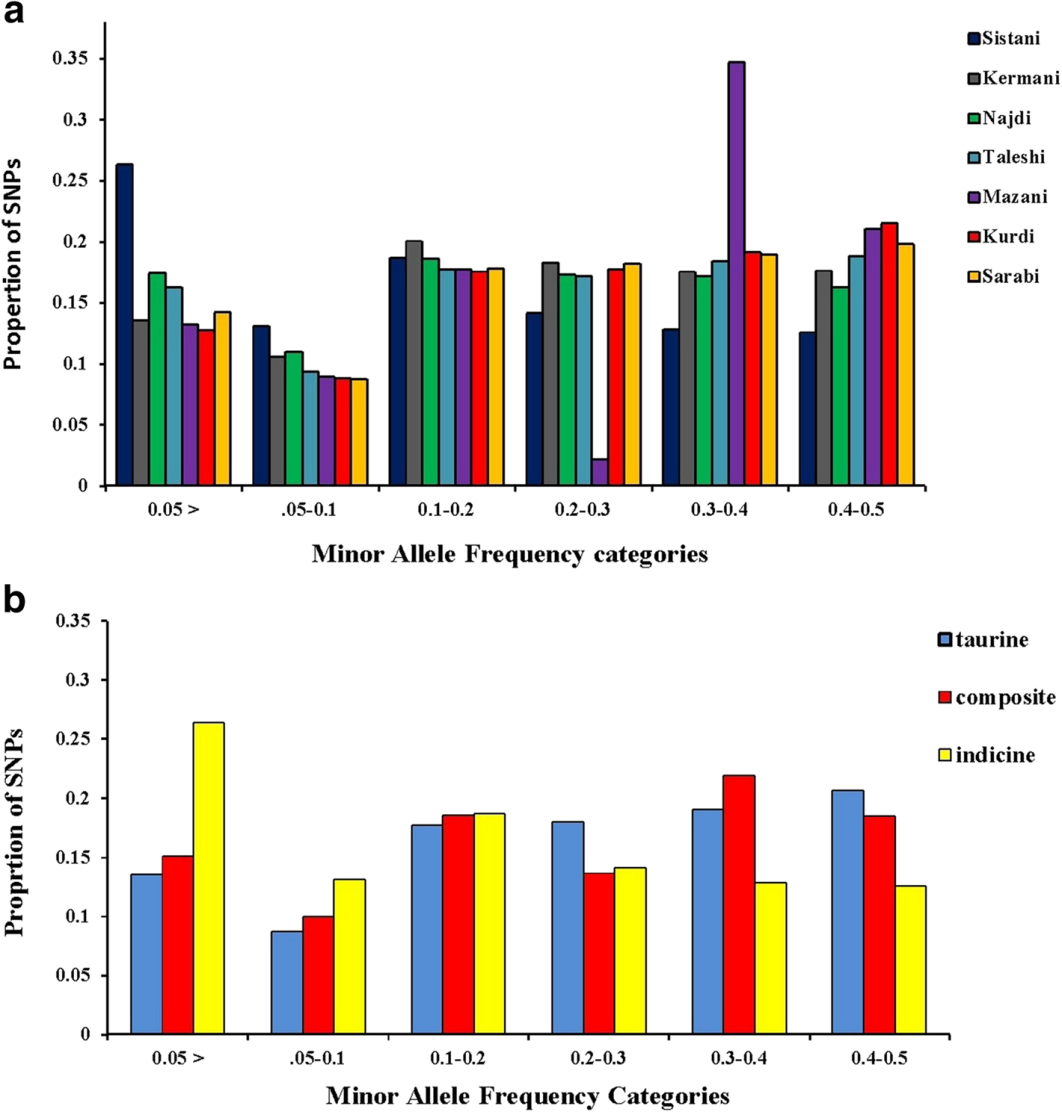
Distribution of minor allele frequencies for (**a**) each Iranian cattle breed and (**b**) for taurine, indicine and composite cattle groups

A total of 70 Iranian native cattle were genotyped for 777962 SNPs. On average, 166742 SNPs remained after quality control, and SNPs had an overall MAF mean of 0.221. Table 1 represented the number of SNPs remained after quality control, overall mean of MAF, average SNP interval (Kb) and total SNP pairwise comparisons in each breed. Figure 1a is given the distribution of the allelic frequencies in different breeds. The highest proportion of SNPs having a MAF less than 0.2 was observed in Sistani breed (58.1 %) while the lowest level of this proportion (39.2 %) was in Kurdi breed. Average proportions of SNPs with MAF < 0.2 in taurine, indicine and composite breeds were equal to 40, 58.1 and 43.8 %, respectively (Fig. 1b).

**Table 1:** Representation of the total number of analyzed SNPs, Average of MAF, average SNP Interval (Kb) and total SNP pairwise comparisons per each breed

Breed	Breed type	Number of samples	Average of MAF	Total analyzed SNPs	Average SNP Interval (Kb)	Total SNP pairwise comparisons
Sistani	Bi	10	0.171	147209	17.1	4179397
Kermani	Bt × Bi	10	0.222	170490	14.6	5562714
Najdi	Bt × Bi	10	0.215	178076	13.9	6077845
Taleshi	Bt × Bi	10	0.226	143246	17.8	3956155
Mazandarani	Bt × Bi	10	0.232	227010	11.1	9836173
Kurdi	Bt	10	0.243	136252	18.2	3776474
Sarabi	Bt	10	0.238	164915	15.1	5305292

**Fig. 2:**
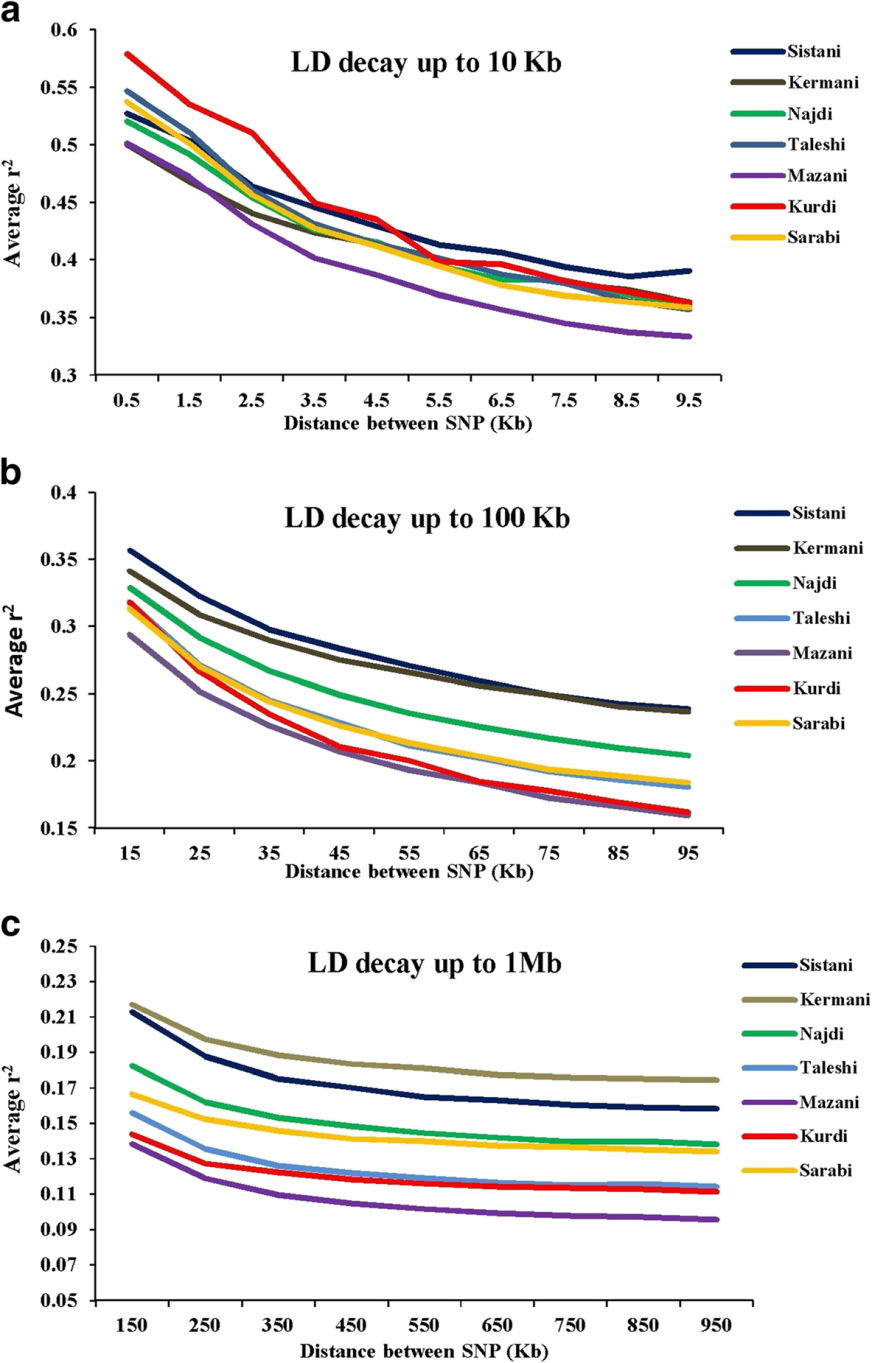
LD decays represented by the average r^2^ for the three SNP sets: SNP pairs separated by inter-marker distances of (**a**) 0 until 10 Kb using consecutive 1 Kb bins (**b**) 10 Kb until 100 Kb using consecutive 10 Kb bins and (**c**) 100 Kb until 1000 Kb using consecutive 100 Kb bins

**Fig. 3:**
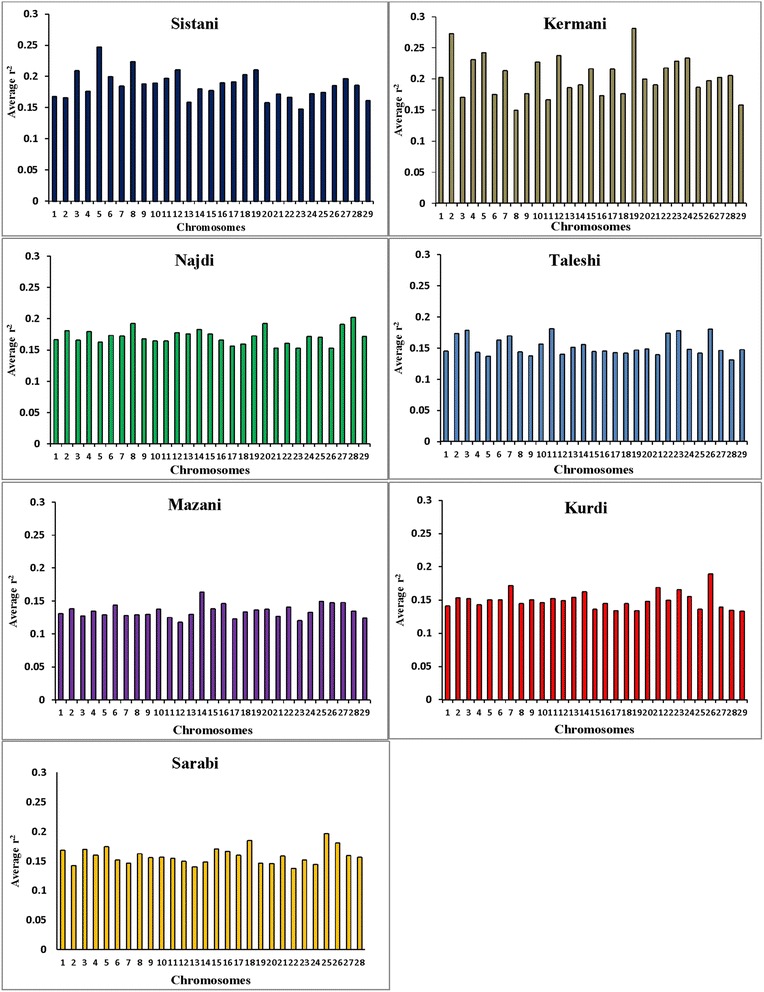
Comparison of mean values of r^2^ per each chromosome (chr:1–29) among different Iranian cattle breeds

Average r^2^ between adjacent markers was estimated for each chromosome within different breeds (Table 2). The pattern of LD was significantly different among various chromosomes in each breed. The highest average r^2^ between adjacent SNP was in the Sistani breed (r^2^ = 0.393) while the lowest average r^2^ was observed in Kurdi aniamls (r^2^ = 0.321). LD means at various intervals were computed by grouping all syntenic SNP combinations using bins of 1 Kb (intervals spanning up to 10 Kb), 10 Kb (intervals spanning 10 Kb to 100 Kb) and 100 Kb (intervals spanning 100 Kb to 1Mb) in whole autosomal chromosomes. Table 3 was represented the statistical information for average r^2^ as distance between SNP pairs up to 500 Kb in each breed. Decay of LD for SNP pairs, categorized in the three distance sets, was represented by the average r^2^ of consecutive bins in Fig. 2 The average r^2^ declines with increasing physical distance between markers in all breeds. However, the degree of fluctuations was different between various populations. Mazandarani cattle had the lowest average LD among other breeds for all the studied distances between markers. Over very short distances between markers (<3 Kb), Kurdi, Taleshi, Sarabi and Sistani breeds had the higher average LD (r^2^ > 0.5) and the Kurdi breed had the highest average LD (r^2^ > 0.53) among others. However, LD decayed faster in Kurdi, Sarabi and Taleshi breeds than Sistani breed in the larger distances between markers (see Fig. 2). While Kermani breed had a lower average r^2^ than most other breeds in the distances less than 3 Kb, LD decay was very slow in this breed such that the highest LD levels were observed in Kermani breed for higher than 100 Kb distances (see Table 3 and Fig. 2). The average r^2^ were different among various autosomal chromosomes in each breed (Fig. 3). Higher LD values were found for BTA5 (r^2^ = 0.21), BTA19 (r^2^ = 0.24), BTA28 (r^2^ = 0.21), BTA11 (r^2^ = 0.14), BTA14 (r^2^ = 0.12), BTA26 (r^2^ = 0.14) and BTA25 (r^2^ = 0.2) in Sistani, Kermani, Najdi, Taleshi, Mazandarani, Kurdi and Sarabi breeds, respectively. This may be attributed to different selection criteria in each breed that have influenced the particular QTLs on different chromosomes.

**Table 2:** Average r^2^ between adjacent SNPs obtained for each autosomal chromosome in seven Iranian cattle breeds

Autosomal chromosome	Breed						
	Sistani	Kermani	Najdi	Taleshi	Mazandarani	Kurdi	Sarabi
1	0.387	0.387	0.367	0.327	0.349	0.304	0.371
2	0.383	0.452	0.407	0.357	0.373	0.349	0.350
3	0.402	0.365	0.361	0.387	0.358	0.316	0.364
4	0.386	0.409	0.382	0.316	0.355	0.311	0.345
5	0.456	0.431	0.366	0.332	0.361	0.323	0.374
6	0.402	0.367	0.375	0.355	0.366	0.337	0.372
7	0.405	0.403	0.393	0.369	0.347	0.358	0.337
8	0.409	0.338	0.401	0.322	0.355	0.317	0.345
9	0.423	0.367	0.370	0.329	0.355	0.328	0.356
10	0.391	0.409	0.376	0.353	0.369	0.323	0.373
11	0.402	0.365	0.382	0.390	0.371	0.355	0.385
12	0.415	0.418	0.376	0.313	0.331	0.313	0.351
13	0.385	0.361	0.366	0.332	0.346	0.320	0.315
14	0.378	0.378	0.384	0.324	0.403	0.332	0.354
15	0.385	0.389	0.375	0.334	0.371	0.290	0.365
16	0.401	0.372	0.394	0.336	0.388	0.326	0.38
17	0.412	0.390	0.353	0.308	0.332	0.288	0.36
18	0.441	0.377	0.367	0.328	0.361	0.325	0.369
19	0.416	0.447	0.373	0.336	0.367	0.305	0.366
20	0.360	0.376	0.408	0.332	0.361	0.325	0.341
21	0.374	0.390	0.374	0.318	0.355	0.347	0.366
22	0.367	0.416	0.367	0.382	0.378	0.332	0.331
23	0.324	0.414	0.335	0.350	0.310	0.323	0.314
24	0.398	0.408	0.373	0.321	0.365	0.323	0.32
25	0.386	0.377	0.385	0.324	0.383	0.281	0.42
26	0.385	0.382	0.354	0.362	0.372	0.380	0.385
27	0.369	0.354	0.373	0.303	0.348	0.287	0.342
28	0.385	0.384	0.4	0.301	0.363	0.294	0.348
29	0.377	0.312	0.343	0.321	0.321	0.287	0.312
Average	0.393	0.388	0.375	0.337	0.359	0.321	0.355

**Table 3:** Average r^2^ values and ± standard deviations over different physical distances, pooled over all autosomes, in seven Iranian cattle breeds

SNP pairs Distance	Breed						
	Sistani	Kermani	Najdi	Taleshi	Mazandarani	Kurdi	Sarabi
0-1 Kb	0.527 ± 0.396	0.499 ± 0.39	0.52 ± 0.387	0.546 ± 0.379	0.501 ± 0.377	0.579 ± 0.375	0.537 ± 0.371
1-2 Kb	0.503 ± 0.399	0.468 ± 0.388	0.491 ± 0.388	0.510 ± 0.379	0.471 ± 0.378	0.535 ± 0.375	0.500 ± 0.374
2-3 Kb	0.464 ± 0.396	0.44 ± 0.386	0.454 ± 0.385	0.461 ± 0.379	0.431 ± 0.375	0.510 ± 0.379	0.456 ± 0.374
3-4 Kb	0.445 ± 0.393	0.423 ± 0.38	0.425 ± 0.379	0.430 ± 0.376	0.401 ± 0.369	0.449 ± 0.381	0.427 ± 0.372
4-5 Kb	0.429 ± 0.391	0.413 ± 0.378	0.415 ± 0.38	0.413 ± 0.377	0.386 ± 0.367	0.434 ± 0.375	0.411 ± 0.371
5-6 Kb	0.413 ± 0.387	0.401 ± 0.376	0.394 ± 0.375	0.401 ± 0.373	0.369 ± 0.364	0.398 ± 0.370	0.394 ± 0.366
6-7 Kb	0.406 ± 0.386	0.387 ± 0.373	0.382 ± 0.37	0.387 ± 0.371	0.357 ± 0.361	0.396 ± 0.371	0.377 ± 0.361
7-8 Kb	0.394 ± 0.381	0.379 ± 0.372	0.382 ± 0.371	0.379 ± 0.371	0.344 ± 0.356	0.381 ± 0.364	0.369 ± 0.360
8-9 Kb	0.385 ± 0.38	0.373 ± 0.369	0.37 ± 0.367	0.364 ± 0.359	0.337 ± 0.354	0.372 ± 0.362	0.363 ± 0.358
9-10 Kb	0.39 ± 0.381	0.363 ± 0.365	0.36 ± 0.364	0.356 ± 0.362	0.333 ± 0.354	0.363 ± 0.362	0.358 ± 0.356
10-20 Kb	0.365 ± 0.372	0.341 ± 0.36	0.329 ± 0.355	0.317 ± 0.348	0.294 ± 0.338	0.317 ± 0.349	0.313 ± 0.343
20-30 Kb	0.322 ± 0.359	0.308 ± 0.347	0.291 ± 0.339	0.271 ± 0.328	0.251 ± 0.317	0.266 ± 0.325	0.269 ± 0.324
30-40 Kb	0.289 ± 0.349	0.289 ± 0.339	0.267 ± 0.327	0.245 ± 0.312	0.226 ± 0.302	0.234 ± 0.310	0.244 ± 0.310
40-50 Kb	0.283 ± 0.342	0.275 ± 0.332	0.249 ± 0.317	0.228 ± 0.304	0.206 ± 0.290	0.210 ± 0.292	0.226 ± 0.300
50-60 Kb	0.271 ± 0.336	0.266 ± 0.327	0.235 ± 0.308	0.211 ± 0.292	0.193 ± 0.280	0.200 ± 0.286	0.213 ± 0.290
60-70 Kb	0.259 ± 0.33	0.256 ± 0.322	0.225 ± 0.302	0.202 ± 0.287	0.184 ± 0.273	0.185 ± 0.273	0.203 ± 0.284
70-80 Kb	0.249 ± 0.324	0.249 ± 0.318	0.217 ± 0.297	0.192 ± 0.278	0.172 ± 0.264	0.177 ± 0.269	0.193 ± 0.276
80-90 Kb	0.242 ± 0.32	0.24 ± 0.314	0.21 ± 0.291	0.185 ± 0.275	0.165 ± 0.259	0.169 ± 0.259	0.189 ± 0.273
90-100 Kb	0.239 ± 0.319	0.236 ± 0.311	0.204 ± 0.288	0.180 ± 0.270	0.159 ± 0.253	0.161 ± 0.253	0.183 ± 0.269
100-200 Kb	0.212 ± 0.301	0.216 ± 0.299	0.182 ± 0.271	0.156 ± 0.249	0.138 ± 0.234	0.143 ± 0.238	0.166 ± 0.255
200-300 Kb	0.187 ± 0.283	0.197 ± 0.299	0.162 ± 0.253	0.135 ± 0.229	0.118 ± 0.213	0.127 ± 0.221	0.152 ± 0.242
300-400 Kb	0.175 ± 0.273	0.188 ± 0.278	0.152 ± 0.245	0.125 ± 0.219	0.109 ± 0.203	0.122 ± 0.216	0.145 ± 0.236
400-500 Kb	0.17 ± 0.269	0.183 ± 0.274	0.148 ± 0.241	0.121 ± 0.214	0.104 ± 0.197	0.118 ± 0.212	0.141 ± 0.232

## Discussion

The LD maps can be used to explore the diversity between cattle breeds with different evolutionary history. In order to compare the extent of LD in the Iranian cattle genome, we analyzed SNP genotyping data belonged to seven cattle breeds. The observed mean r^2^ values were significantly different between Iranian cattle populations. This can be reflected different population history, selection pressures and inbreeding levels in each breed. The average r^2^ for SNPs less than 1 Kb apart was found to be equal to 0.579 (Kurdi), 0.546 (Taleshi), 0.537 (Sarabi), 0.527 (Sistani), 0.52 (Najdi), 0.501 (Mazandarani) and 0.499 (Kermani) in this study. Furthermore, the mean r^2^ ranged from 0.387 (Mazandarani) to 0.435 (Kurdi) for the SNPs less than 10 kb apart. The LD measure for SNPs up to 1 kb apart have been reported to be equal to 0.34 (Nellore cattle) [26], 0.55-0.75 (several Bos taurus and Bos indicus cattle breeds) [16] and 0.767 (Australian Holstein-Friesian) [27]. The average r^2^ values ranging from 0.25 (Brahman) to 0.49 (Hereford) were reported by Porto-Neto et al. [28] in eight cattle breeds at < 10 kb distances between markers. Moreover, Mokry et al. [29] found similar LD range (0.25–0.40) at short distances (<10 kb) between markers for Brazilian composite beef cattle breeds. The LD decay was also analyzed for distances from 10 Kb up to 100 Kb using the 10 Kb bins. These results show that average r^2^ have been started at 0.294–0.356 range (10–20 Kb bin) and reached to 0.159– 0.239 range (90–100 Kb bin) for Iranian cattle breeds. Salomon-Torres et al., [15] investigated the LD levels in 19 cattle breeds. They reported that for distances from 95 Kb up to 100 Kb, the lowest averages of LD were in Piedmontese (0.085), Sheko (0.104) and Charolais (0.105) while the highest average of LD were in Hereford (0.222), Jersey (0.201), and Brown Swiss (0.177) breeds. Additionally, ranges of average r^2^ were equal to 0.11–0.23 (Nellore) [26], 0.153-0.402 (Australian Holstein-Friesian) [27], 0.13-0.27 (Canchim) [29] and 0.16-0.30 (Chinese Simmental) [19]. These results confirmed that the logical ranges of average r^2^ were obtained for the two first studied distance sets.

The mean r^2^ declined more slowly with increasing physical distances between markers for distances larger than 100 Kb and was almost constant after 500 Kb of distance. After 100 Kb up to 1 Mb, average r^2^ ranged from 0.11 to 0.216 among various defined bins in Iranian cattle LD data. Comparing to the other studies conducted on indigenous Swiss cattle (0.06–0.14) [18], Australian Holstein-Friesian (0.057–0.108) [27], Canchim (0.07–0.1) [29] and Chinese Simmental (0.05-0.08) [19], it appears that r^2^ values were overestimated in this study for larger distances (>100 Kb). Khatkar et al. [2008] [27] pointed out that the studies involved relatively small sample sizes are subject to bias and loss of accuracy and this bias may vary with inter-marker distance. Certainly, small sample sizes have influenced the r^2^ values obtained for Iranian cattle breeds in this study. However, r^2^ values estimated at the shorter distances, have more reliability and can be used to compare LD levels. The main idea of this study was based on representation a general picture to compare the LD levels between Iranian cattle breeds. However, any comparison with other studies should be conservative. LD levels have been generally influenced by factors such as sample size, MAF thresholds, density of SNP panels and distance markers among different studies [19, 30]. Despite the sample size bias, it would be reasonable to expect extensive LD in Iranian cattle populations. Iranian indigenous cattle breeds have been included small populations that were exposed to serious extinction risk in recent years. Population decline, increasing inbreeding and uncontrolled crossbreeding are of concerns, and it should be given more attention to conserve these genetic resources.

Much more LD fluctuations were observed in the distances less than 10 Kb among different breeds. After 10 Kb apart between markers, LD decay had the similar trend in most of the breeds. Kermani breed continued to have more slowly LD decay than all of the other breeds after 3 Kb distances. It appears that population decline has been initiated earlier in Kermnai than other breeds. More rapid LD decay was observed in Kurdi breed for distances less than 100 Kb than other breeds. However, LD decay has been slower in Kurdi breed for the larger distances indicating the rising trend of population decline in more recent generations for this population. It appears that Sarabi breed has also had more intense population decline in recent times. Among the studied breeds, Mazandarani had the lowest LD level in different intervals of genome which could be an indicator of larger ancestral population in this breed. Based on both phenotype characteristics and genetic structure analysis, Sistani breed has more indicine genetic background [31]. Results of the previous studies [15, 16] indicated that LD levels were less in indicine breeds. However, in this study, Sistani breed (as a indicine breed) had higher LD level among other studied breeds. This can be attributed to historical smaller effective population sizes [25] or a higher ancestral relatedness [32] in Sistani breed. Extensive LD variability was observed among different chromosomes that can probably be evidence on varying recombination rates, selection effects and genetic drift between chromosomes [14]. Meuwissen et al. [5] suggested that the LD levels should be above 0.2 to achieve an accuracy of 0.85 for genomic breeding values. Useful LD to give sufficient power for genome-wide-association studies (GWAS) have been suggested to be above 0.3 [1, 33]. Average distance between markers ranged from 11.1 Kb (Mazandarani) to 18.2 Kb (Kurdi) among Iranian cattle populations in this study. Average r2 above 0.2 was given at distances less than 200 Kb (Sistani and Kermani), 100 Kb (Najdi), 70 Kb (Taleshi and Sarabi), 60 Kb (Kurdi) and 50 Kb (Mazandarani) in our study. In other hand, the average r2 above 0.3 was observed at distances less than 30 Kb for Sistani and Kermani, 20 Kb for Najdi, Taleshi, Kurdi and Sarabi, and 10 Kb for Mazandarani.

Different LD patterns on individual chromosomes among various breeds could be created through uneven selection pressures on QTLs distributed throughout the genome. So, higher LD can be expected for chromosomes harboring quantitative trait loci (QTL) undergoing selection [34, 35]. Number of significant detected QTLs located on some chromosomes was explored for several important traits of interest in Iranian cattle breeds using two QTL databases available online (http://www.animalgenome.org/cgi-bin/QTLdb/BT/index. and http://bovinegenome.org/bovineqtl_v2/login.jsp) (Table 4). This investigation confirmed that certain chromosomes presented higher LD in each breed have included more numbers of QTLs pertaining to important traits attributed to that breed (see Fig. 3 and Table 4). In accordance with presence of QTLs identified for some traits such as growth, carcass weight, meat percentage, body height, resistance to clinical mastitis and calf size on the chromosome 5, higher average r^2^ was also seen for this chromosome in Sisatni breed (that is popular for mentioned traits). Also, it appears that several traits such as milk fat, somatic cell score and udder attachment have been affected by stronger selection in Kermani breed (chromosome 19). Mazandarani breed has some prominent traits such as growth efficiency, carcass weight, tick resistance and high milk fat and protein that can be largely explained by QTLs located on chromosome 14. In agreement with higher average r^2^ found for chromosome 26 in Kurdi breed, more QTLs associated with prominent traits of Kurdi breed (such as small body size, Calving ease and milk related traits) were also observed for this chromosome. Similar trend can be reported for QTLs on chromosome 11 that were related to clinical mastitis, SCC and milk fat in Taleshi breed. More selections on calving ease, calf size, dairy capacity and milk protein in Sarabi breed may have led to higher average r^2^ found for genomic regions located on chromosome 25. Furthermore, chromosome 28 and 8 have included QTLs affecting traits highly attributed to Najdi cattle such as easy calving, Immunoglobuin G level, heat tolerance and high milk protein. In harmony with these findings, lower LD levels were also observed for chromosomes containing lower numbers of QTLs identified for outstanding traits of Iranian cattle (Table 4). However, LD levels can be depended on some other factors such as recombination rates, mutation rates, genetic drift and population size. Hence, a more detailed study on selected regions of the genome are required and assessing signatures of positive selection can be suggested for future investigations.

**Table 4:** Number of detected QTLs on each chromosome for some important traits in cattle breeds^a^

Traits	Chromosomes														
	2	5	8	11	12	14	18	19	22	23	25	26	27	28	29
Average daily gain	0	1	1	2	0	6	0	0	3	0	0	2	0	0	4
Meat percentage	1	3	0	0	0	0	0	0	0	0	0	0	0	0	0
Body weight (mature)	2	2	1	2	4	1	3	3	1	2	0	2	2	1	2
Height (mature)	0	3	5	0	3	3	0	0	1	1	1	3	1	0	1
Carcass weight	7	4	4	3	2	9	0	0	2	3	2	2	3	2	5
Calving ease	2	2	5	1	3	3	8	5	3	4	7	4	1	2	0
Calf size	0	2	0	0	3	0	6	3	0	0	6	2	0	0	2
Clinical mastitis	0	8	0	4	0	1	0	1	0	0	0	1	0	0	0
Somatic cell score	2	5	2	5	3	7	8	5	2	2	2	1	3	1	1
Heat tolerance	0	0	1	0	3	0	0	0	1	1	1	1	1	0	0
Dairy capacity	0	0	0	1	1	1	0	0	0	0	1	0	0	0	0
Udder attachment	0	0	0	0	0	0	2	4	1	4	0	3	0	1	0
Dry matter intake	1	1	0	1	0	0	1	0	0	2	0	0	0	0	0
Insemination per conception	0	4	0	0	0	3	0	1	0	0	1	0	0	0	0
Milk fat traits	2	7	0	2	5	8	5	4	2	1	1	10	0	0	2
Milk protein traits	2	11	7	6	2	14	7	4	4	7	7	6	3	6	0
Milk yield	3	7	1	4	4	8	4	1	1	5	1	4	2	3	0
Tick resistance	1	2	0	1	0	1	0	0	0	2	0	0	1	0	0
Immunoglobuin G level	1	0	4	1	0	3	3	0	0	2	0	0	0	1	0
Total detected QTLs	149	175	88	110	93	163	124	168	87	84	84	119	74	57	97

^a^Only significant QTLs were reported

In this study, large number of SNPs were excluded from the data set due to lack of enough quality. This may be attributed to DNA quality, however the GC-score, which provides information on the genotyping quality, did not show any abnormal deviations (16 % of markers had a GC score <0.5), and therefore we don’t believe that there have been issues with DNA quality. SNP ascertainment bias effect must be acknowledged in this study. Iranian indigenous cattle have not been included in the bovine genome sequencing projects and the SNPs on the chip have been mainly selected based on information from European taurine cattle (also refrence genome is from a taurine breed), this can be somewhat affected genotyping quality. However, the number of remained SNPs was enough to perform analysis and could provide more information, for instance, compared to 50 K chips. Despite of removing a large part of SNP data due to low quality, these results confirmed that the SNP densities used in this work can provide enough accuracy for the future genomic selection programs and GWAS in Iranian cattle.

## Conclusions

A wide genetic diversity and different historical background were well reflected in LD levels among Iranian cattle breeds. LD fluctuations were more detectable in the shorter distances (less than 10 Kb) among different breeds. Despite of the sample size effects, High LD levels obtained in this study confirmed the small size of Iranian cattle populations that were exposed to serious extinction risk in recent years.
